# Equine Herpesvirus Type 4 (EHV-4) Outbreak in Germany: Virological, Serological, and Molecular Investigations

**DOI:** 10.3390/pathogens10070810

**Published:** 2021-06-25

**Authors:** Selvaraj Pavulraj, Kathrin Eschke, Jana Theisen, Stephanie Westhoff, Gitta Reimers, Sandro Andreotti, Nikolaus Osterrieder, Walid Azab

**Affiliations:** 1 Institut für Virologie, Robert von Ostertag-Haus, Zentrum für Infektionsmedizin, Freie Universität Berlin, Robert-von-Ostertag-Str. 7–13, 14163 Berlin, Germany; pselvaraj1@lsu.edu (S.P.); k.eschke@fu-berlin.de (K.E.); no.34@fu-berlin.de (N.O.); 2 Die Mobile Pferdepraxis, Haberkamp 3, 22927 Großhansdorf, Germany; theisen@mobile-pferdepraxis.com (J.T.); westhoff@mobile-pferdepraxis.com (S.W.); reimers@mobile-pferdepraxis.com (G.R.); 3 Department of Mathematics and Computer Science, Institute of Computer Science, Freie Universität Berlin, Arnimallee 14, 14195 Berlin, Germany; Sandro.Andreotti@fu-berlin.de; 4 Department of Infectious Diseases and Public Health, Jockey Club College of Veterinary Medicine and Life Sciences, City University of Hong Kong, Kowloon Tong 999077, Hong Kong

**Keywords:** EHV-4, herpesvirus, equine, outbreak, clinical signs, respiratory disease, diagnosis

## Abstract

Equine herpesvirus type 4 (EHV-4) is enzootic in equine populations throughout the world. A large outbreak of EHV-4 respiratory infection occurred at a Standardbred horse-breeding farm in northern Germany in 2017. Respiratory illness was observed in a group of in-housed foals and mares, which subsequently resulted in disease outbreak. Out of 84 horses in the stud, 76 were tested and 41 horses were affected, including 20 foals, 10 stallions, and 11 mares. Virological investigations revealed the involvement of EHV-4 in all cases of respiratory illness, as confirmed by virus isolation, qPCR, and/or serological follow-up using virus neutralization test and peptide-specific ELISA. Among infected mares, 73% (8 out of 11) and their corresponding foals shed the virus at the same time. EHV-4 was successfully isolated from four animals (including one stallion and three foals), and molecular studies revealed a different restriction fragment length polymorphism (RFLP) profile in all four isolates. We determined the complete 144 kbp genome sequence of EHV-4 isolated from infected horses by next-generation sequencing and de novo assembly. Hence, EHV-4 is genetically stable in nature, different RFLP profiles, and genome sequences of the isolates, suggesting the involvement of more than one animal as a source of infection due to either true infection or reactivation from a latent state. In addition, epidemiological investigation revealed that stress caused by seasonal changes, management practices, routine equestrian activities, and exercises contributed as a multifactorial causation for disease outbreak. This study shows the importance of implementing stress alleviating measures and management practices in breeding farms in order to avoid immunosuppression and occurrence of disease.

## 1. Introduction

Infections with equine herpesviruses (EHV) are widespread in equine populations throughout the world. Nine herpesviruses have been identified so far: six belong to the subfamily *Alphaherpesvirinae* (EHV-1, 3, 4, 6, 8, and 9) and three belong to *Gammaherpesvirinae* (EHV-2, 5, and 7) [1]. EHV-1 and EHV-4, the important pathogens among other equine herpesviruses, belong to the genus *Varicellovirus*. Both viruses are closely related genetically and antigenically with considerable cross reactivity [2,3]. EHV-1 causes respiratory infection, abortion, neonatal foal mortality, and equine herpesvirus myeloencephalopathy [4,5]. EHV-4 causes only moderate respiratory infection in foals and horses of less than 2 years old. In addition, EHV-4 infections are mostly inapparent without any clinical signs, and the infections in foals and horses may go unnoticed [6]. As described earlier, EHV-4 is endemic in equine populations, and serological surveys indicate seroprevalence of more than 80% among horses, donkeys, and mules in different geographical locations [7,8,9,10,11,12]. The higher seroprevalence shows the evidence of heavy exposure to the virus; however, epidemiological data regarding source and time of infection of naïve animals is not available. It is generally accepted that EHV-4 infection starts early in life, where foals and yearlings are the most clinically affected.

Following infection and a short incubation period, animals may develop pyrexia, anorexia, mandibular lymphadenopathy, dry cough, and serous to mucopurulent nasal discharge [13]. Mostly, infected animals may recover within two to three weeks after infection. EHV-4 undergoes latency in trigeminal ganglion of infected animals with periodic reactivation [14,15]. Horses are repeatedly infected by EHV-4 in nature or by reactivation from latency; however, disease signs are less severe when compared to EHV-1, with subsequent episodes in later life. EHV-4 can infect peripheral blood mononuclear cells (PBMC) and endothelial cells [16,17]. However, unlike EHV-1 [18], PBMC-associated viremia, abortions, and neurological impairment due to EHV-4 are rare.

Diagnosis of EHV-4 infection/shedding is commonly based on PCR or quantitative PCR (qPCR) analysis of nasal swabs/washes [13]. Serological-based assays, such as the virus neutralization test (VNT), plaque reduction, and peptide-based ELISA assays, are also available. Acute and convalescent serum samples with significant increase in antibody titer (four-fold) can indicate recent infection [19,20,21]. In addition, virus isolation on primary cell culture system is considered the gold standard assay, which facilitates further comparative and molecular analyses. Further, restriction fragment length polymorphism (RFLP) and whole genome sequencing are used to identify genetic variations among virus isolates using phylogenetic analysis [3,22].

Vaccination against viral infections is the only mean of disease prevention. There are two commercial EHV-4 vaccines available; i.e., Flu-Vac Innovator^®^ 6, which protects against six common equine pathogens, including EHV-1 and 4, and Equivac innovator^®^ EHV1/4, which protects against both EHV-1 and 4. However, recent randomized control studies that have assessed the efficacy of commercial inactivated combined EHV-1/EHV-4 vaccines have shown that vaccination did not reduce the respiratory illness and viremia but reduced virus shedding and abortion [23,24]. It is worthy to mention that data regarding protective efficacy against EHV-4, in comparison to EHV-1, are fewer and require further investigations. Along with vaccination, implementation of vigorous hygienic measures can also help in reducing virus spread between horses.

Here, we report a typical outbreak pattern of EHV-4 with clinical signs and rapid spread of infection within a breeding farm with subsequent virus isolation and characterization. The present study describes clinical, virological, serological, and molecular findings of respiratory infection caused by EHV-4 in a breeding farm in Germany.

## 2. Results

### 2.1. The Outbreak

**Figure 1:**
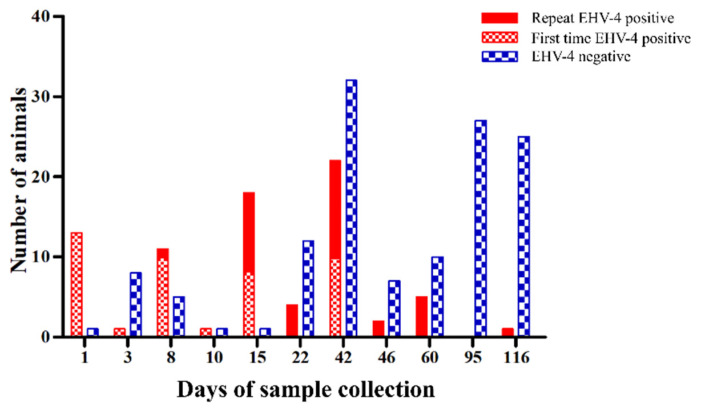
Equine herpesvirus type 4 (EHV-4) disease outbreak pattern in the breeding farm. The number of quantitative PCR first time positive animals, repeated EHV-4 positive, and EHV-4 negative animals during the period of sample collection following the outbreak is shown.

During July 2017, an outbreak of a respiratory illness was reported in a group of foals and mares at a breeding farm in northern Germany. It is noteworthy that prior to the outbreak, three foreign breeding mares were introduced to the farm. During the first week of the outbreak, 12 foals in a group of 25 foals showed clinical signs of mild to moderate respiratory illness. Molecular investigation by qPCR revealed EHV-4 infection in 11 out of the 12 foals. At the same time, two corresponding mares housed with the foals had mild respiratory signs and were tested positive for EHV-4. Clinical signs in foals were largely restricted to the upper respiratory tract, characterized by pyrexia, cough, nasal discharge, and anorexia. The disease course in infected foals lasted for 7 to 14 days. Immediately after diagnosis, biosecurity measures were applied, and management practices were implemented. Biosecurity measures to curtail the outbreak included movement restrictions, assigning different personnel for handling sick animals, and the use of disinfectant foot baths and hand sanitizers. Further, healthy animals that had already contacted the sick animals were monitored daily for clinical signs. However, EHV-4 infected horses were not separated from healthy horses due to insufficient space to keep all infected horses in isolation pens. Infected and healthy horses shared common housing and feeding facilities in the farm throughout the period of disease outbreak. Nasal swabs were regularly collected from infected and contact animals to identify any new infected animals and to study the outbreak pattern. Despite biosecurity measures, during the second and third weeks after the start of the outbreak, the disease spread to a nearby barn, and several foals and mares showed mild to moderate respiratory illness and were confirmed EHV-4-positive. Disease outbreak peaked between the second and eighth weeks after detecting the first EHV-4-positive case in the farm and lasted for 17 weeks (Figure 1; Table 1). Some apparently healthy mares also tested positive for EHV-4 by qPCR and shed viruses through nostrils continuously for up to 8 weeks. It was surprising that some mares/stallions shed the virus through nostrils for a long time (i.e., Mare_3, Stallion_6; Table 1) without showing any clinical signs despite the mares being vaccinated against EHV-1 during the third, fifth, and eighth months of pregnancy, in addition to the regular annual vaccination. None of the unweaned foals were vaccinated before or at the time of the outbreak. In total, nasal swabs were collected from 76 animals including 25 foals, 15 stallions, 34 mares, and 2 geldings at different intervals (Table 1; foal numbers given in the table correspond to their mare numbers, e.g., foal 1 was born to mare 1 and so on). Infected individuals (foals, mares, and stallions) were tested maximum at five time points and minimum at two time points before they tested negative for EHV-4 (Table 1). Ages of infected foals ranged between 85 and 209 days with average of 125.5 ± 32 days (Table 1). Among infected mares, 73% (8/11) of mares and their corresponding foals shed the virus at the same time. Most of the infected foals were in physical contact with other foals, mares, and/or stallions, as groups were built for social contact very early (2–3 weeks after birth), which suggests direct contact as the mode of virus transmission during later phase of disease outbreak.

### 2.2. Virus Shedding

Nasal swabs were collected from foals, stallions, and mares throughout the course of disease outbreak at different time points (Table 1). Virus shedding through nostrils was investigated using qPCR. A cycle threshold (CT) value of ≤39 was considered as positive for EHV-4 shedding. In total, 80% (20/25) foals, 67% (10/15) stallions, and 32% (11/34) mares tested positive for virus shedding. Out of EHV-4 positive animals, 54% (22/41) of horses shed viruses through nostrils (in nasal swabs) at more than one time point during the outbreak. The remaining 46% of the animals showed virus shedding once, and no EHV-4 viral DNA was detected in nasal swabs in later time points (Table 1). Fisher’s exact test showed significant (*p* < 0.05) higher rates of infection and virus shedding in foals and yearling stallions in comparison to mares. In addition, 75% of the infected foals showed virus shedding for 14 days on an average with typical course of EHV-4 infection, initially shedding high virus load (low CT values) followed by low virus load (high CT values), and then the animal became negative for EHV-4 nucleic acids. However, some foals shed the virus for 6 to 9 weeks. In general, foals and mares with low CT values shed viruses for longer durations than those with high CT values. Differences in clinical signs among those animals could not be correlated based on CT values. A few foals (e.g., Foal_1, Table 1) became completely negative for virus shedding 2 weeks after initial infection, but 6 weeks later, showed secondary virus shedding with low CT values (CT = 23.9; 26.2) without any clinical signs.

### 2.3. Virus Isolation

**Figure 2:**
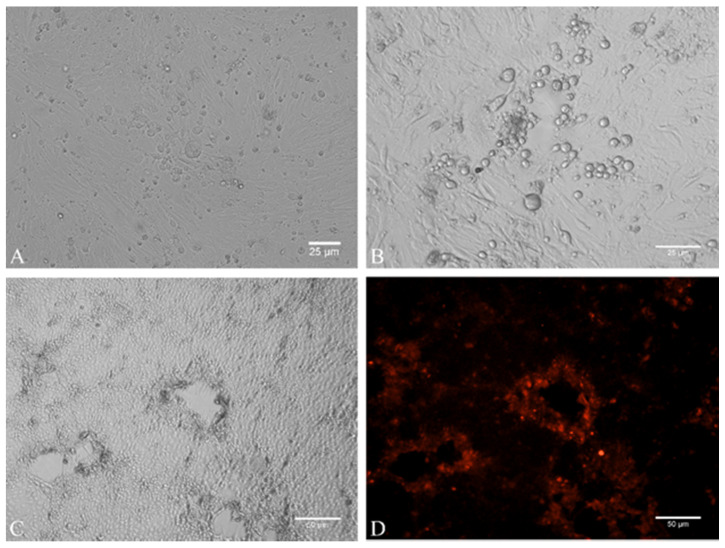
Equine herpesvirus type 4 (EHV-4) isolation in equine dermal (ED) cells. (**A**–**C**) Cytopathic effect caused by EHV-4 on ED cells characterized by rounding of cells and syncytia formation at 24 h post infection. (**D**) Indirect immunofluorescence staining of EHV-4-infected cells from (C) to detect glycoprotein D (gD) expression in EHV- at 24 h post infection. Red: gD stained with primary anti-gD antibodies and secondary Alexa Fluor-568 antibody.

Virus isolation from infected animals (mares, stallions, and foals) has been attempted on equine dermal (ED) primary cells. Nasal swabs with low CT values (<25; *n* = 12; from seven foals, four stallions, and one mare) and moderate CT values (25–30; *n* = 4; from two foals and two mares) were selected. In total, EHV-4 was isolated from four animals including one stallion (Stallion_6: EHV-4_DE17_1) and three foals (Foal_11: EHV-4_DE17_2; Foal_13: EHV-4_DE17_3; Foal_4: EHV-4_DE17_4). In all four cases, cytopathic effect characterized by rounding of the cells, syncytia formation, and detachment started to appear 48 h post inoculation in passage 1 (Figure 2A–C). It is noteworthy to mention that virus isolation was successful from the four nasal swabs, which had low CT values (high virus titer) (Table 1). The virus could not be isolated from nasal swabs with moderate CT values (CT ≥ 25) even after five blind passages on ED cells. Indirect IF confirmed EHV-4 specific gD expression in infected ED cells (Figure 2D).

### 2.4. Serology

**Figure 3:**
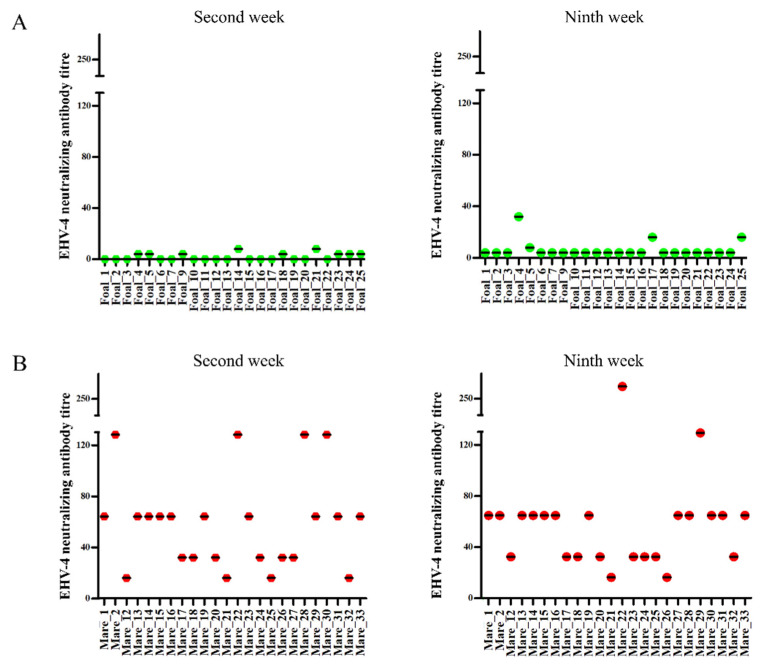
Virus neutralization assay. Equine herpesvirus type 4-(EHV-4) specific virus neutralizing antibody titer was determined at the time of the outbreak [Second week] and after the outbreak period [Ninth week] in foals (**A**) and mares (**B**), respectively. Green dots: foals; Red dots: mares.

**Figure 4:**
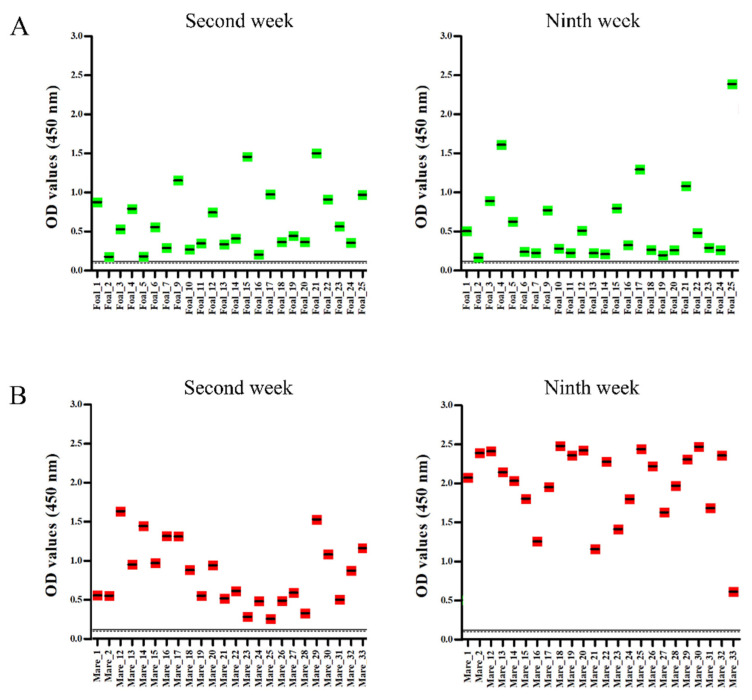
Equine herpesvirus type 4 (EHV-4) glycoprotein G (gG)-peptide based-enzyme-linked immunosorbent assay. Average optical density (OD) values of antibodies against gG of EHV-4 at the time of the outbreak [Second week] and post outbreak period [Ninth week] were determined in foals (**A**) and mares (**B**), respectively. Green dots: foals; Red dots: Mares. Continuous and dotted lines show the cut-off values for questionable and negative results for EHV-4 antibodies, respectively.

VNT and EHV-4 gG-peptide based-ELISA were performed on paired serum samples collected from 24 mares and their corresponding 24 foals. VNT revealed that none of the foals had neutralizing antibodies against EHV-4 at the time of the outbreak (2nd week), and only three foals (12.5%) were subsequently seroconverted at the 9th week (with non-protective antibody titer, <64) (Figure 3A,B; Table 2). In contrast, all mares had neutralizing antibodies at the time of the outbreak (antibody titer of >8; all mares were seropositive), and 58.33% of mares had protective antibody titer (≥64) against EHV-4. None of the mares were seroconverted (no four-fold increase in antibody titer) at the end of the outbreak. On the other hand, peptide ELISA assay showed that all tested foals and their corresponding mares had specific antibody against gG of EHV-4 at the time of the outbreak (Figure 4A,B; Table 2) and persisted until the end of disease outbreak without major changes. Serological response to EHV-4 infection determined by gG peptide ELISA was compared with standard virus neutralization test. Mares already had EHV-4 specific neutralizing antibodies in serum, which might be due to previous infection and/or vaccination. All foals had detectable EHV-4 specific antibodies in serum as determined by peptide ELISA; however, only three foals had neutralizing antibodies, which could be due to delayed onset of neutralizing antibody production and/or interference of maternally-derived antibodies.

### 2.5. RFLP Analysis

**Figure 5:**
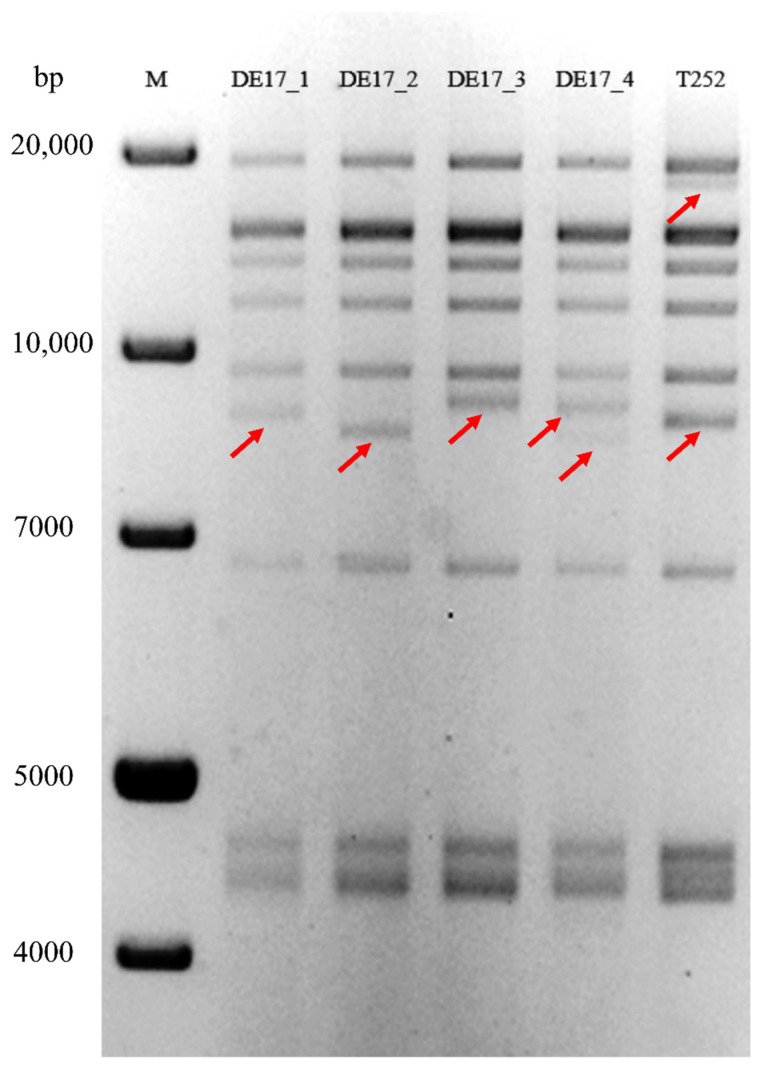
Restriction fragment length polymorphism (RFLP) analysis for equine herpesvirus type 4 (EHV-4) isolates. The BamH1 digestion of genomic DNA from the four isolate of EHV-4 (DE17_1-4) and reference isolate (T252) is shown. For RFLP, 1.5 µg of viral DNA was digested with BamH1 for 4 h at 37 °C and separated on 0.8% agarose gel by electrophoresis at 60 Volt for 16 hr. M—marker (1 kb plus DNA ladder [Thermo Fisher Scientific^TM^, Waltham, MA, USA]). Red arrows indicate differences in DNA fragment size. bp—base pair.

The BamH1 digestion of genomic DNA from the four isolates of EHV-4 (DE17_1-4) revealed four different restriction patterns. By comparing the restriction profiles obtained in this study, including the reference isolate (T252), we were able to conclude that all four isolates were distinct from each other (Figure 5). The digestion profiles of the isolates were different from those of the reference strain due to the presence of different size fragments, approximately between 8 and 9 kb. Isolate 1 (DE17_1) had a fragment at 8.5 kb, isolate 2 (DE17_2) had a fragment at 8 kb, isolate 3 (DE17_3) had a fragment at 9 kb, and isolate 4 (DE17_4) had two bands at 8.5 and 9 kb positions. RFLP prediction with the available whole EHV-4 genome (Genbank accession: KT324742.1 [virus isolate from Australia] and NC_001844.1 [virus isolate from United Kingdom]) revealed that the fragment of the 8.5 kb size was between genome sequences of 42,867 and 51,555 bp size, which code, partially, very large tegument proteins, including repeat regions (ORF24: 36,006–46,610 bp), capsid protein (ORF25: 47,068–46,610 bp), membrane associated phosphoprotein (ORF26: 47,980–47,156 bp), DNA packaging proteins (ORF27 and 28: 48,543–50,365 bp), uncharacterized protein (ORF29: 50,358–51,338 bp), and partially, DNA polymerase protein (ORF30: 54,924–51,262 bp). Differences in the restriction profile reflect changes in the sequence of genes listed above, confirmed by whole genome sequencing analysis that revealed differences in sequence length of repeat regions associated with the C-terminal sequence of the very large tegument protein. Furthermore, none of the four isolates had a fragment of approximately 18 kb, which was only observed with the reference virus strain (T252).

### 2.6. Whole Genome Sequencing

The datasets of the four samples comprised between ~1.7 and 2.2 million paired-end reads, of which between ~144 and ~230 thousand remained after mapping against the pan-genome. Mapping assemblies against the ~144 kb reference genome LC075586.1 resulted in average coverages between ~164 and ~282. The numbers of reference bases with missing consensus due to low coverage or low quality (miraconvert with threshold 20) were between 50 and 119. The sequence gap was closed by PCR amplification of missing sequences using DNA from EHV-4 isolates as DNA template. Amplified PCR products were sequenced, and the resulting sequences were integrated into the corresponding EHV-4 genome assemblies. The final 144 kb complete genome sequences for all four isolates were obtained and submitted to Genbank (Genbank accession numbers: MW892435, MW892436, MW892437 and MW892438).

### 2.7. Phylogenetic Analysis

**Figure 6:**
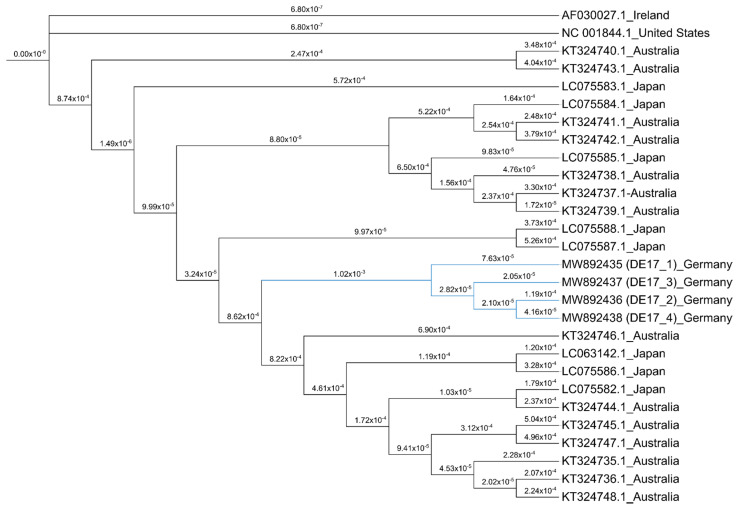
Phylogenetic tree constructed by the maximum-likelihood method using full genome sequences of the four equine herpesvirus type 4 (EHV-4) isolates and other EHV-4 reference genomes. GenBank accession numbers are indicated with country of origin. EHV-4 sequences from the current study are indicated in blue.

A phylogenetic tree was constructed for the four full genome sequences of EHV-4 isolates, as well as other reference genomes (Figure 6). For analysis, we used complete genomes of EHV-4; 14 Australian isolates, eight Japanese isolates, and one isolate from Ireland and USA. The four EHV-4 isolates from the current study clustered together and were closely related to isolates from Australia and Japan (99.7% and 99.85% homology, respectively).

Whole ORF30 gene and partial gB gene of EHV-4 isolates were successfully PCR amplified. By sequencing of ORF30 and gB genes, all four of our isolates possessed the same nucleotide sequence. All sequence obtained for ORF30 and gB showed almost 99% similarity with EHV-4 sequences published in Genbank. Phylogenetic analysis for ORF30 was performed on 22 isolates and strains of EHV-4 including the four German isolates from the current study, 10 isolates from Australia, six isolates from Japan, and one isolate from USA and Ireland. They were clustered into two groups, I and II (Supplementary Figure S1A). The genome sequences of the 23 isolates and strains appeared to be very similar, especially in group II. Our four German isolates clustered into group I. Our isolates were closely related to viruses from Australia and Japan. The structure of the gB tree was constructed on 19 isolates (four German isolates (from the current study), two Ireland isolates, one USA isolate, six Australian isolates and six Japanese isolates). The gB tree clustered all four German isolates into group II and the remaining 15 isolates into group I (Supplementary Figure S1B). All the 15 isolate sequences available online, as mentioned earlier, had the same gB sequence, but our four isolates had a single nucleotide change at position 624 (61280 of genome) from T to C. However, biological relevance and significance of this point mutation on gB need to be studied. In addition, phylogenetic analysis for gG was performed on 20 isolates and strains of EHV-4, including the four German isolates from the current study, seven isolates from Australia, six isolates from Japan, and one isolate from USA, United Kingdom, and Ireland. Our four isolates clustered with the viruses from Ireland, Japan, and Australia (Group I; Supplementary Figure S1C).

## 3. Discussion

The present study reports a large outbreak of respiratory infection and subsequent detection and isolation of EHV-4 from affected foals and their corresponding mares and stallions in a breeding stud farm in northern Germany. Three main factors may have initiated the outbreak: including (i) introducing three breeding mares with unknown history of EHV-1 or EHV-4 status to the farm, (ii) seasonal changes, and (iii) weaning. During the first week of July 2017, a group of foals and mares began to show signs of respiratory infection. Immediate qPCR analysis of nasal swabs confirmed EHV-4 infection in almost all foals with respiratory signs. All foals tested negative for EHV-1. Clinical signs were mild to moderate in foals and almost inapparent in mares and stallions, with a few exceptions where pyrexia, dyspnea, and nasal discharge were observed. This is in agreement with previous reports [25] where most of the clinically infected horses were less than three years of age, and the majority of affected horses were foals and weanlings, and nearly all EHV-4 positive aged horses were healthy. Biosecurity measures were implemented subsequently; however, they did not mitigate the outbreak completely, and more horses were infected and experienced respiratory illness and shed the virus during the second and third weeks of the outbreak. This could be because of the fact that most of the foals in the farm already contracted the infection from corresponding mares or from silent shedders (mares and stallions) and began to show clinical signs during the second and third weeks. However, this does not exclude that the biosecurity measures prevented further spread of infection outside the stud and helped other animals not to contract the infection.

One interesting finding of the study was the different restriction digestion (RFLP) pattern of the four isolates that might indicate that more than one virus was circulating in the farm at the time of the outbreak. It is suggested that EHV-4 is highly stable genetically in comparison to EHV-1 [26], so the concept of possible mutations during the outbreak can be excluded. Further, it suggests that there was no single animal source (index cases) for disease outbreak.

Generally, foals were kept with their corresponding mares until weaning. Most of the EHV-4 positive foals in the current study were either unweaned or weaned recently. Foals from which the virus was isolated were unweaned during the outbreak. In addition, most of the foals and their corresponding mares tested positive for EHV-4. It is possible that EHV-4 was reactivated from a latent state in mares due to the above-mentioned stressful factors and resulted in infection of their corresponding foals and other foals from negative mares or stallions in contact. Although mares were positive for EHV-4 by qPCR, CT values were very low, indicating virus shedding at very low level. Our virus isolation trials also raise the concern about CT values, as virus was successfully isolated from nasal swabs with CT values of less than 25. No virus was isolated from samples with CT values above 25, even after five blind passages. Animals with low CT values shed viruses through nostrils for an extended period of time (up to 9 weeks) in comparison to animals with high CT values. A few mares shed viruses at a few early time points, became negative for a few weeks before beginning to shed the virus again, which indicates the occurrence of intermittent reactivation in silent shedders and necessitates a long isolation period for infected animals and frequent sample collection and testing before declaring the animal is EHV-4 negative. Significance of this very low virus shedding for long periods and its role in disease outbreak would be a very interesting aspect to study in the future. In addition, regular virological surveillance for EHV-4 in mares during gestation and until a few weeks after weaning would give a clear picture about EHV-4 reactivation and the transmission cycle from mares to foals and may be helpful in identifying and formulating suitable intervention strategies to prevent disease spread.

Horses are considered long day seasonal breeders. Period of foaling is restricted to few weeks in the year and most of the foals are the same age on average. During the time of weaning, most of the foals may not have maternally-derived antibodies, and weaning stress makes the foals susceptible to infection. In our case, weaning coincided with seasonal changes (severe rain in summer). Meanwhile, yearlings in stud were already broken and were housed closely with broodmares and foals. A limited number of feeding spaces for horses in the paddock is also a factor attributed to stress. Horses in different stages were routinely conglomerate with each other during routine farm activities. In stressed mares and stallions, EHV-4 virus might have been reactivated and spread to foals by direct and indirect means. This shows the importance of stress in disease outbreak and necessitates exercising stress-alleviating measures. Previous reports suggest that EHV-4 infection may happen throughout the year [27]; however, the current study indicates the impact of the season in inducing stress and subsequent disease outbreak. Biosecurity measures play a major role in the control of disease outbreaks. Restricting the entry of horses displaying clinical signs of disease, following specific quarantine measures and testing for common diseases for new horses, and separating horses in the facility according to activity and age are essential measures. Continuous and regular monitoring of health status for all equids on the premises should always be applied. The stall should be constructed in a way to curtail disease transmission, especially with non-porous walls and floors. All stalls should be disinfected and cleared of bedding after each use. In addition, immunizing horses with vaccines containing EHV-4 would be an ideal strategy to prevent or reduce the incidence of the outbreak. However, vaccine efficacy in controlling EHV-4 associated disease outbreak needs further studies [23,24].

In most cases, EHV-4 causes inapparent infections, even in foals, as reported previously [28]. Nevertheless, in our study, we observed distinct clinical signs in most of the infected foals and in some mares. This could be because of differences in the virus strains and/or regular monitoring and surveillance of the animals in the farm for presence of any clinical signs. Although EHV-4 can infect PBMCs and endothelial cells in vitro [17], and abortions have been reported to be caused by EHV-4 in mares [29,30,31], no abortions and mortalities were observed in any of the infected animals during the outbreak.

As reported earlier, it is clear from our study that foals were infected in early age without distinct seroconversion, as evidenced by our serological assays. The average age of infected foals was 125.5 ± 32 days and ranged between 85 and 209 days. A few foals had low levels of EHV-4 neutralizing antibodies (titer of 1:4–1:8) and gG specific antibodies at the time of outbreak. These could be maternally-derived antibodies as most of the foals were unweaned. Foals did not have protective neutralizing antibodies against EHV-4 at the time of disease outbreak, which indicates that foals were highly susceptible to EHV-4 infection around the weaning time, as maternally-derived antibody levels in serum go down. In contrast, all mares had EHV-4 neutralizing antibodies in the serum due to prior infection and/or immunization. Only 12.5% of infected foals were seroconverted at the end of the outbreak. None of the mares were seroconverted, which shows that mares with protective antibody titer can develop inapparent infection and shed the virus without seroconversion. Furthermore, no clear correlation could be established between levels of neutralizing antibodies and severity of clinical sings. As mentioned earlier, few foals had low level of antibodies against EHV-4 without seroconversion; this could be because of residual maternally-derived antibodies, as reported earlier [32,33]. Although foals tested negative for neutralizing antibodies using VNT, EHV-4 gG specific antibodies could be detected through peptide ELISA. Therefore, peptide ELISA can be used as an effective tool for detecting antibodies against EHV-4 during early infection and VNT for late infection in foals.

All four EHV-4 isolates had similar ORF30, gG and gB gene sequence and different RFLP pattern. The isolated virus strains were different from previously reported existing strains. ORF-30 and gG sequences from our study mostly clustered with EHV-4 isolates from Australia and Japan. Sequence analysis of gB gene revealed a unique point mutation in position T624 to C624, which clustered our four isolates into a separate group. Importance of this point mutation in gB and its role in virus biology need to be studied.

We determined the whole 144 kb genome sequence of the EHV-4 isolates by next-generation sequencing and de novo assembly. EHV-4 genome analysis of our current study confirms the relatedness of the virus to other reported whole genome sequences of EHV-4. Furthermore, whole genome sequencing results of all four virus isolates identified the genetic diversity of the viruses, which further supports simultaneous involvement of multiple mares in the outbreak.

In the current study, we aimed to investigate the clinical, virological, serological, and molecular findings of a respiratory infection caused by EHV-4 in a breeding stud farm in Germany. However, a few potential limitations were anticipated. (i) As we have confirmed EHV-4 infection based on clinical and molecular investigation and ruled out EHV-1 infection, we have not investigated other possible bacterial, viral, and parasitic causes of respiratory illnesses. (ii) It was difficult to have access to clinical samples of all animals at each time point, as once the animal became negative, clinical samples were not being collected. (iii) Clinical data of some individual animals could not be accessed at certain time points. (iv) It was out of the scope of this study to confirm the assumption of reactivation from latency by specific assays.

## 4. Materials and Methods

### 4.1. Premise and Horses

In July 2017, an outbreak of EHV-4 occurred in an equine breeding farm in northern Germany. The affected breeding farm housed Standardbred horses with three stallions, 28 pregnant mares, 13 non-pregnant mares, 18 yearlings (12 males and six females), 13 fillies, and 12 colts at the time of outbreak. The breeding farm also housed 36 racehorses in a nearby separate paddock, which remained unaffected by EHV-4 during the period of disease outbreak in main farm. Mares and corresponding foals were divided into four groups with seven mares and foals in each group ranged by date of foaling. If a broodmare lost a foal, she was sent to the non-pregnant group of mares when tested negative for EHV-1 and EHV-4. Mares and corresponding foals remained in the same box till weaning. Horses were routinely vaccinated against EHV-1 (Prevaccinol^®^, MSD AnimalHealth, Kenilworth, NJ, USA; attenuated live vaccine), equine influenza (ProteqFlu^®^, Boehringer Ingelheim, Ingelheim am Rhein, Germany; canarypox vectored vaccine, H3N8), and tetanus as per manufacturer’s instructions. Mares were vaccinated against EHV-1 three times during pregnancy at the third, fifth and eighth months. Yearlings were vaccinated twice a year after weaning.

In 2012, the same breeding farm experienced a severe EHV-1 outbreak with respiratory tract infection, neurological illness, and abortion [10]. In 2015, all yearlings were sick with fever and nasal discharge, starting on March 21 and ended on April 14. However, all animals tested negative for EHV-1 and EHV-4. Yearlings (born in 2016) started the outdoor season in April 2017 (males and females). When nights were frost free, older foals and mares stayed in the grass day and night. They were only brought inside for health checkups one or two times a week or when needed for other reasons. In between March and May 2017, two mares aborted, and one foal died at birth on day 319 of pregnancy; however, they tested negative for EHV-1 and EHV-4. In June 2017, three foreign mares from two different owners (without vaccination) entered the breeding farm for breeding without quarantine. Those three breeding mares were transported in a transport box that had been regularly used for mares, foals, and racehorses transport. Weaning of the foals started on 5 October 2017 and ended on 6 November 2017. Foals aged between 150 and 200 days old were weaned. During the outbreak, foals stayed with the corresponding mares. It is noteworthy to mention that in June and July 2017, there was intermittent heavy rain alternating with warm summer weather at farm location.

Foals infected in the current EHV-4 outbreak have never been vaccinated against EHV-1 and EHV-4. The outbreak of EHV-4 occurred following the onset of respiratory illness in foals and mares. Infected foals showed pyrexia, cough, and nasal discharge. However, a few foals and most of the mares did not show apparent clinical signs.

### 4.2. Sample Collection

On 7 July 2017, two foals began to show signs of respiratory illness and tested positive for EHV-4 in nasal swabs. On subsequent days, several foals showed similar clinical signs. Nasal swabs were collected from mares, yearlings, and foals (*n* = 76) showing signs of respiratory illness, including cough, nasal discharge, and pyrexia, at different time points (Table 1). In some cases, nasal swabs were collected from apparently healthy foals in contact with infected horses, as many foals needed to be tested for EHV-4 status before they traveled to Sweden to obtain passports. Collected nasal swabs in virus transport medium (phosphate buffered saline (PBS) with 2% fetal bovine serum (FBS), 1% penicillin-streptomycin (P-S), and 5 µg/mL of Amphotercin B (Biochrom^TM^ GmBH, Berlin, Germany)) were transported to the diagnostic laboratory, Institute of Virology, Freie Universität Berlin, at 4 °C for subsequent analysis. Paired serum samples were collected from selected infected animals (*n* = 48; 24 mares and 24 corresponding foals) at the second and ninth weeks of disease outbreak. The period of EHV-4 outbreak was between 7 June and 17 November 2017 and lasted for 133 days.

### 4.3. Quantitative (q)-PCR

Total viral DNA were extracted from (200 µL) nasal swabs using the RTP DNA-RNA virus mini kit (Stratec Molecular GmbH^®^, Birkenfeld, Germany) as per manufacturer’s instructions. qPCR was performed with StepOnePlus^TM^ Real-time PCR system (Applied Biosystems, Foster City, CA, USA). Primers (Fwd 5′-CGCAGAGGATGGAGACTTTTACA-3′ and Rev 5′-CATGACCGTGGGGGTTCAA-3′) and probes (5′FAM-CTGCCCGCCGCCTACTGGATC-TAMRA) specific to glycoprotein B (gB) gene of EHV-4 were used as described previously [34]. The 20 µL of the reaction mixture [5 µL of extracted DNA, 10 µL SensiFAST™ Probe Lo-ROX (2x) (Meridian Bioscience, Cincinnati, OH, USA), 10 pmoli/µL of probe (0.9 µL), 10 pmoli/µL of forward and reverse primers (0.9 µL each), and 3 µL of nuclear free water] for each sample were analyzed. The cycling conditions for thermal profile was: hold for 2 min (min) at 95 °C, 40 cycles of amplification (3 s (sec) at 95 °C and 30 sec at 60 °C with data collection) and hold for 1 min at 60 °C for data collection. Positive (DNA extracted from EHV-4-infected ED cells culture supernatant) and negative (nuclease free water) extractions were performed and included in every run. Nasal swab samples were considered negative for EHV-4 if the CT values were >39.

### 4.4. Cell Culture and Virus Isolation

Virus isolation was attempted for the qPCR-positive nasal swab samples using ED cells. The cells were grown in Iscove’s modified Dulbecco’s medium (IMDM, Pan^TM^, Biotech, Aidenbach, Germany) supplemented with 20% FBS (Pan^TM^, Biotech, Aidenbach, Germany), 1% non-essential amino acids (Biochrom^TM^ GmBH, Berlin, Germany), 1 mM Sodium pyruvate (Pan^TM^, Biotech, Aidenbach, Germany), and 1% P-S. Nasal swabs in virus transport medium were vortexed and centrifuged at 6000× *g* for 5 min. The supernatant was collected, 2% P-S and 5 µg/mL of Amphotercin B were added, and it was incubated for 30 min at 4 °C. Meanwhile, ED cells were trypsinized and suspended at a concentration of 3 × 10^5^ cells/mL. In 24-well plate, each 100 µL of the prepared supernatant was mixed with 400 µL of ED cell suspension and incubated at 37 °C. Cells were examined daily till day 5 for the presence of cytopathic effect (CPE). If no CPE observed in the first passage, inoculated cells were subjected to five more blind passages.

### 4.5. Indirect Immunofluorescence Assay (Indirect-IF)

Indirect-IF was performed to detect EHV-4 specific viral antigen in cell culture. Briefly, ED cells were grown in a 24-well plate and infected with 50 plaque forming units (PFU) of the isolated EHV-4 viruses. After 48 h (hrs) post infection, cells were fixed with 4% paraformaldehyde (PFA) and incubated for 30 min at room temperature (RT). Fixed cells were permeabilized with 0.1% Triton X-100 for 5 min and blocked with 3% bovine serum albumin (BSA; VWR^®^ life science; Radnor, PA, USA) for 1 hr. Cells were incubated overnight with primary EHV-4 anti-glycoprotein D monoclonal antibody (kindly provided by Dr Jules Minke, Merial, Lyon, France; dilution: 1:400) at 4 °C [35]. After washing, cells were probed with goat anti-mouse IgG (H + L) labeled with Alexa fluor-568 (A11019, Invitrogen^®^, Carlsbad, CA, USA; dilution: 1:500) for 1 hr. Mock infected cells were stained with the same dilutions of primary and secondary antibodies. Plates were analyzed using Zeiss^®^ Axio Vert.A1 fluorescence microscope (Carl Zeiss AG, Jena, Germany).

### 4.6. Virus Neutralization Test (VNT)

Virus neutralization test was performed, according to the OIE reference protocol [21], to evaluate the virus neutralizing antibody titer against EHV-4 in the collected serum samples. Briefly, serum samples were heat inactivated at 56 °C for 30 min. Reference EHV-4 strain T252 [36] propagated in ED cells was diluted in minimum essential medium (MEM, Pan^TM^ Biotech, Aidenbach, Germany) to obtain 450–600 PFU/mL. In 96-well plate, 25 µL of test serum samples were serially two-fold diluted till 1:512 in MEM. Similarly, positive, and negative control sera were added in respective wells. The virus (25 µL of working concentration; 45–65 PFU/4 wells) was added to each well, and the plates were incubated at 37 °C for 1 hr. Trypsinized ED cells (50 µL; 3 × 10^5^ cells/mL) were added to each well and incubated at 37 °C for 1 h. Finally, 100 µL of 1.6% carboxy methyl cellulose medium (Dulbecco’s MEM (Biochrom^TM^ GmBH, Berlin, Germany), 5% FBS, and 1% P-S) was added to all wells and incubated at 37 °C. After 72 h, cells were fixed and stained with 0.5% crystal violet. Virus neutralizing antibody titer was calculated by determining the reciprocal of the highest serum dilution that caused 50% of plaque number reduction. Antibody titer of ≤4 was considered negative, 8–32 was positive but non-protective, and ≥64 antibody titer was positive and protective against infection. Furthermore, a four-fold increase in titer between paired sera was considered seroconversion.

### 4.7. Peptide ELISA

EHV-4 glycoprotein G (gG) based peptide enzyme linked immunesorbent assay (ELISA) was performed to assess serum antibody response against EHV-4 during and after the disease outbreak. The assay was performed as described before [20]. The assay was repeated three independent times for each serum sample. The average optical density (OD) values of ≥0.118 were considered positive, OD values between 0.100 and 0.118 were considered questionable, and OD values of <0.100 were considered negative.

### 4.8. Restriction Fragment Length Polymorphism (RFLP)

RFLP analysis was performed for genomic characterization of EHV-4 isolates. Viral DNA was extracted from the four EHV-4 isolates and the reference EHV-4 laboratory strain (T252) using Sinzger method [37]. Briefly, EHV-4 full-infected ED cells were incubated with a cell permeabilization buffer (1.28 mol/L sucrose, 20 mmol/L MgCl_2_, 40 mmol/L Tris-HCl, and 4% Triton X-100; pH 7.5) on ice for 10 min and centrifuged at 1300× g for 15 min at 4 °C. The pellet was resuspended in equal volumes of cell nuclei buffer (10 mmol/L Tris-HCl; pH 7.5, 2 mmol/L MgCl_2_, and 10% sucrose) and 2× nuclease buffer (40 mmol/L PIPES; pH 7.0, 7% sucrose, 20 mmol/L NaCl, 2 mmol/L CaCl_2_, 10 mmol/L 2-mercaptoethanol, and 0.2 mmol/l PMSF) with 2000 Gel Units micrococcal nuclease (New England Biolabs, Ipswich, MA, USA) and incubated for 30 min at 37 °C. Digestion buffer (100 mmol/L NaCl, 10 mmol/L Tris-HCl; pH 8.0, 25 mmol/L EDTA; pH 8.0, 0.5% SDS, and 0.1 mg/mL Proteinase K) with 0.2 mol/l EDTA was added, and the lysate was incubated overnight at 50 °C. Viral DNA was extracted by phenol/choloroform/isoamyl alcohol. A total of 0.5 volume of 7.5 mol/L ammonium acetate and 2 volume of absolute ethanol were added to the aqueous phase. DNA pellets were washed with 70% ethanol and resuspended in Tris-EDTA buffer. The obtained DNA was stored at 4 °C till use. For RFLP, 1.5 µg of viral DNA was digested with BamH1 (New England Biolabs, Ipswich, MA, USA) for 4 h at 37 °C and separated on 0.8% agarose gel.

### 4.9. Lllumina Library Preparation and Sequencing

For next generation sequencing (NGS) library preparation, viral DNA was extracted from ED cells infected with all four EHV-4 isolates using the innuPREP Virus DNA/RNA Kit (AnalytiK Jena^TM^, Überlingen, Germany). Total DNA (5 µg) was diluted in 130 µL TE buffer and fragmented to a peak fragment size of 500 bp using the Covaris M220 focused-sonicator with appropriate settings. The resulting DNA fragments (fragment size of 500–700 bp) were gel-purified after 1% agarose gel electrophoresis for size selection. The purified DNA was subsequently used to generate Illumina libraries using the NEBNext Ultra II Library Prep Kit for Illumina platforms (New England Biolabs, Ipswich, MA, USA) according to the manufacturer’s instruction. To complete the adaptor sequences and to achieve a library yield >500 ng, eight PCR cycles were performed at the end of the protocol. The index-amplified libraries were quantified using NEBNext Library Quant Kit for Illumina (New England Biolabs, Ipswich, MA, USA) and a StepOnePlus^TM^ Instrument (Applied Biosystems, Foster City, CA, USA). Following quantification, samples were pooled to equimolar amounts to achieve a library concentration of 4 nM. The library pool was diluted further to load a final amount of 16 pM onto an Illumina MiSeq machine (Illumina Inc., Hayward, CA, USA) for DNA sequencing.

### 4.10. Sequencing Data Analysis

NGS read data was used for genome assembly of the viral genomes [38]. Reads were filtered by mapping against a pan genome sequence (BWA-MEM [39] version 0.7.17) built from 22 whole genome assemblies of EHV-4; only read pairs with at least one read mapping the pan genome were used for final assembly. Initial assemblies were produced by mapping against the reference genome LC075586.1 using the mapping assembly mode of MIRA [40] (version 4.9.6). As the resulting assemblies had missing sequences, the sequence gap was closed by designing specific primer sequences (fwd-CATCCACAGTTTCACCAACACC and rev-GTCATCATCTGGTAGGGGAGTG) and the subsequent PCR amplification of missing sequence using DNA from EHV-4 isolates as a DNA template. Amplified PCR products were sequenced, and resulting sequences were integrated into corresponding EHV-4 genome assemblies, and final sequence was obtained.

### 4.11. Genome Sequencing and Phylogenetic Analysis

For clustering of EHV-4 isolates, sequencing of partial gB gene and complete ORF30 (DNA polymerase) gene was performed for all four isolates using specific primer sets; gB—gB4fwd CATGTCTAAAGACTCGACAT and gB4rev CGCAAACCATAATACCAATC; ORF30—AATCTCGAGTCAGCTTTGATGGGGAACTG and AGAACTGCCCAGTGTGAAGG; ACCCCCTTCATGAGCAT and GGAGGGCTGTTTAAGGTCTG; ATACAATACTCTCCTATTAC and ATTGCGGCCGCATGGCGGCGCACGAACAGGA; and AGCAAACCGCGACGGGTCGT and ATTGCGGCCGCATGGCGGCGCACGAACAGGA. PCR products were purified using the GF-1 AmbiClean kit^®^ (Vivantis Technologies, Selangor, Malaysia) and sequenced (LGC Biosearch Technologies, Hoddesdon, UK). EHV-4 glycoprotein G (gG) gene sequences obtained from the whole genome sequencing analysis were also used for phylogenetic analysis. Maximum-likelihood phylogenetic tree was constructed by aligning the nucleotide sequences of the isolated four whole EHV-4 genomes and reference sequences retrieved from Genbank using MEGA7.0.26 software. One thousand bootstrap replicates were used to assess the significance of the tree topology. In addition, phylogenetic analysis of ORF30, gB, and gG of our isolates and reference sequences were performed independently.

### 4.12. Statistics

All results of qPCR, plaque reduction, and ELISA assays were performed in triplicates, and results were interpreted as average values ± standard deviation. Differences in rate of infection among stallions, mares, and foals were analyzed using Fisher’s exact test.

## 5. Conclusions

In conclusion, several horses have harbored, most probably, different EHV-4 infections, as evidenced by RFLP and whole genome sequencing, that resulted in EHV-4 outbreak among foals, mares, and stallions. The source of infection could be attributed to either true infection or reactivation of EHV-4 from latently infected horses.

## Figures and Tables

**Table 1:** Quantitative PCR (qPCR) was performed on DNA extracted from nasal swab samples collected from horses at different time points. Details of animals and qPCR results for equine herpesvirus type 4 in nasal swab samples collected were interpreted. y-Years; m-Months; d-days.

S.No	Animal	Sex	Age	Day 1	Day 3	Day 8	Day 10	Day 15	Day 22	Day 42	Day 46	Day 60	Day 95	Day 116
1.	Mare_1	Mare	12 y 4 m	+	n/a	n/a	n/a	n/a	n/a	+	n/a	n/a	(−)	(−)
2.	Foal_1	Stallion	164 d	++	n/a	n/a	n/a	++	n/a	(−)	n/a	+++	n/a	(−)
3.	Foal_2	Stallion	95 d	+++	n/a	n/a	n/a	+	n/a	+	n/a	n/a	n/a	(−)
4.	Foal_3	Mare	85 d	+/−	n/a	n/a	n/a	+/−	n/a	(−)	n/a	n/a	n/a	n/a
5.	Foal_4	Stallion	126 d	+++ ^a^	n/a	n/a	n/a	+	n/a	+/−	n/a	n/a	n/a	(−)
6.	Mare_2	Mare	12 y 7 m	+	n/a	n/a	n/a	n/a	n/a	+	n/a	n/a	(−)	n/a
7.	Foal_5	Mare	154 d	+++	n/a	n/a	n/a	++	n/a	++	n/a	n/a	n/a	(−)
8.	Foal_6	Stallion	121 d	++	n/a	n/a	n/a	+	n/a	+	n/a	n/a	n/a	(−)
9.	Foal_7	Mare	87 d	+	n/a	n/a	n/a	+	n/a	(−)	n/a	n/a	n/a	n/a
10.	Foal_8	Mare	95 d	+	n/a	n/a	n/a	n/a	n/a	+	n/a	n/a	n/a	(−)
11.	Foal_9	Stallion	87 d	+/−	n/a	n/a	n/a	+/−	n/a	(−)	n/a	n/a	n/a	n/a
12.	Foal_10	Mare	99 d	(−)	n/a	n/a	n/a	n/a	n/a	(−)	(−)	n/a	n/a	n/a
13.	Foal_11	Mare	106 d	++++ ^a^	n/a	n/a	n/a	++	n/a	(−)	n/a	n/a	n/a	n/a
14.	Foal_12	Mare	120 d	+++	n/a	n/a	n/a	+	n/a	(−)	n/a	n/a	n/a	n/a
15.	Mare_3	Mare	1 y 3 m	n/a	+	+	n/a	++	+	n/a	+	+	n/a	(−)
16.	Stallion_1	Stallion	4 y 4 m	n/a	(−)	n/a	n/a	n/a	n/a	n/a	n/a	n/a	n/a	n/a
17.	Gelding_1	Gelding	5 y 4 m	n/a	(−)	n/a	n/a	n/a	n/a	n/a	n/a	n/a	n/a	n/a
18.	Gelding_2	Gelding	5 y 2 m	n/a	(−)	n/a	n/a	n/a	n/a	n/a	n/a	n/a	n/a	n/a
19.	Mare_4	Mare	2 y 2 m	n/a	(−)	n/a	n/a	n/a	n/a	n/a	n/a	n/a	n/a	n/a
20.	Mare_5	Mare	3 y 2 m	n/a	(−)	n/a	n/a	n/a	n/a	n/a	n/a	n/a	n/a	n/a
21.	Stallion_2	Stallion	3 y 3 m	n/a	(−)	n/a	n/a	n/a	n/a	n/a	n/a	n/a	n/a	n/a
22.	Mare_6	Mare	3 y 3 m	n/a	(−)	n/a	n/a	n/a	n/a	n/a	n/a	n/a	n/a	n/a
23.	Stallion_3	Stallion	3 y 3 m	n/a	(−)	n/a	n/a	n/a	n/a	n/a	n/a	n/a	n/a	n/a
24.	Stallion_4	Stallion	1 y 4 m	n/a	n/a	+/−	n/a	n/a	(−)	n/a	(−)	(−)	n/a	n/a
25.	Stallion_5	Stallion	1 y 4 m	n/a	n/a	+	n/a	n/a	(−)	n/a	(−)	(−)	n/a	n/a
26.	Stallion_6	Stallion	1 y 3 m	n/a	n/a	++++ ^a^	n/a	n/a	+	n/a	+/−	+	(−)	n/a
27.	Stallion_7	Stallion	1 y 3 m	n/a	n/a	+	n/a	n/a	+	n/a	(−)	(−)	n/a	n/a
28.	Stallion_8	Stallion	1 y 3 m	n/a	n/a	+	n/a	n/a	(−)	n/a	n/a	n/a	n/a	n/a
29.	Stallion_9	Stallion	1 y 3 m	n/a	n/a	++++	n/a	n/a	(−)	+	(−)	n/a	n/a	n/a
30.	Stallion_10	Stallion	1 y 2 m	n/a	n/a	++++	n/a	n/a	+	(−)	n/a	n/a	n/a	n/a
31.	Stallion_11	Stallion	1 y 2 m	n/a	n/a	+	n/a	n/a	(−)	(−)	(−)	(−)	n/a	n/a
32.	Stallion_12	Stallion	1 y 2 m	n/a	n/a	+++	n/a	n/a	(−)	n/a	n/a	n/a	n/a	n/a
33.	Stallion_13	Stallion	1 y 2 m	n/a	n/a	++++	n/a	n/a	(−)	n/a	(−)	(−)	n/a	n/a
34.	Mare_7	Mare	1 y 3 m	n/a	n/a	(−)	n/a	n/a	(−)	n/a	n/a	n/a	n/a	n/a
35.	Mare_8	Mare	1 y 3 m	n/a	n/a	(−)	n/a	n/a	(−)	n/a	n/a	n/a	n/a	n/a
36.	Mare_9	Mare	1 y 3 m	n/a	n/a	(−)	n/a	n/a	(−)	n/a	n/a	n/a	n/a	n/a
37.	Mare_10	Mare	1 y 4 m	n/a	n/a	(−)	n/a	n/a	(−)	n/a	n/a	n/a	n/a	n/a
38.	Mare_11	Mare	1 y 3 m	n/a	n/a	(−)	n/a	n/a	(−)	n/a	n/a	n/a	n/a	n/a
39.	Foal_13	Stallion	114 d	n/a	n/a	n/a	++++ ^a^	n/a	n/a	(−)	n/a	n/a	(−)	(−)
40.	Foal_14	Mare	74 d	n/a	n/a	n/a	(−)	(−)	n/a	(−)	n/a	(−)	n/a	n/a
41.	Foal_15	Stallion	147 d	n/a	n/a	n/a	n/a	+	n/a	+	n/a	++	n/a	(−)
42.	Foal_16	Stallion	161 d	n/a	n/a	n/a	n/a	+	n/a	+/−	n/a	(−)	n/a	n/a
43.	Foal_17	Stallion	209 d	n/a	n/a	n/a	n/a	+++	n/a	+	n/a	+/−	n/a	(−)
44.	Foal_18	Stallion	87 d	n/a	n/a	n/a	n/a	++	n/a	(−)	n/a	n/a	n/a	n/a
45.	Foal_19	Mare	95 d	n/a	n/a	n/a	n/a	+	n/a	n/a	n/a	n/a	n/a	(−)
46.	Foal_20	Stallion	99 d	n/a	n/a	n/a	n/a	++	n/a	+/−	n/a	n/a	n/a	(−)
47.	Foal_21	Stallion	100 d	n/a	n/a	n/a	n/a	+	n/a	(−)	n/a	n/a	n/a	n/a
48.	Mare_12	Mare	12 y 5 m	n/a	n/a	n/a	n/a	n/a	n/a	+	n/a	n/a	(−)	(−)
49.	Mare_13	Mare	11 y 4 m	n/a	n/a	n/a	n/a	n/a	n/a	(−)	n/a	n/a	(−)	(−)
50.	Mare_14	Mare	18 y 4 m	n/a	n/a	n/a	n/a	n/a	n/a	+	n/a	n/a	(−)	(−)
51.	Mare_15	Mare	13 y 3 m	n/a	n/a	n/a	n/a	n/a	n/a	+	n/a	n/a	(−)	+
52.	Mare_16	Mare	11 y 6 m	n/a	n/a	n/a	n/a	n/a	n/a	(−)	n/a	n/a	(−)	(−)
53.	Mare_17	Mare	7 y 2 m	n/a	n/a	n/a	n/a	n/a	n/a	(−)	n/a	n/a	(−)	(−)
54.	Mare_18	Mare	6 y 3 m	n/a	n/a	n/a	n/a	n/a	n/a	(−)	n/a	n/a	(−)	(−)
55.	Mare_19	Mare	15 y 3 m	n/a	n/a	n/a	n/a	n/a	n/a	(−)	n/a	n/a	(−)	(−)
56.	Mare_20	Mare	10 y 3 m	n/a	n/a	n/a	n/a	n/a	n/a	(−)	n/a	n/a	(−)	(−)
57.	Mare_21	Mare	5 y 4 m	n/a	n/a	n/a	n/a	n/a	n/a	(−)	n/a	n/a	(−)	(−)
58.	Mare_22	Mare	7 y 3 m	n/a	n/a	n/a	n/a	n/a	n/a	+	n/a	n/a	(−)	(−)
59.	Mare_23	Mare	8 y 4 m	n/a	n/a	n/a	n/a	n/a	n/a	+/−	n/a	n/a	(−)	n/a
60.	Mare_24	Mare	13 y 3 m	n/a	n/a	n/a	n/a	n/a	n/a	+	n/a	n/a	(−)	(−)
61.	Mare_25	Mare	6 y 2 m	n/a	n/a	n/a	n/a	n/a	n/a	+	n/a	n/a	(−)	n/a
62.	Mare_26	Mare	5 y 5 m	n/a	n/a	n/a	n/a	n/a	n/a	(−)	n/a	n/a	(−)	n/a
63.	Mare_27	Mare	13 y 5 m	n/a	n/a	n/a	n/a	n/a	n/a	(−)	n/a	n/a	n/a	n/a
64.	Mare_28	Mare	10 y 3 m	n/a	n/a	n/a	n/a	n/a	n/a	+/−	n/a	n/a	(−)	n/a
65.	Mare_29	Mare	10 y 3 m	n/a	n/a	n/a	n/a	n/a	n/a	(−)	n/a	n/a	(−)	n/a
66.	Mare_30	Mare	9 y 2 m	n/a	n/a	n/a	n/a	n/a	n/a	(−)	n/a	n/a	(−)	n/a
67.	Mare_31	Mare	11 y 2 m	n/a	n/a	n/a	n/a	n/a	n/a	(−)	n/a	(−)	(−)	n/a
68.	Mare_32	Mare	7 y 5 m	n/a	n/a	n/a	n/a	n/a	n/a	(−)	n/a	n/a	(−)	n/a
69.	Mare_33	Mare	16 y 4 m	n/a	n/a	n/a	n/a	n/a	n/a	+/−	n/a	(−)	(−)	n/a
70.	Foal_22	Mare	154 d	n/a	n/a	n/a	n/a	n/a	n/a	(−)	n/a	n/a	n/a	n/a
71.	Foal_23	Mare	103 d	n/a	n/a	n/a	n/a	n/a	n/a	(−)	n/a	n/a	n/a	n/a
72.	Foal_24	Mare	100 d	n/a	n/a	n/a	n/a	n/a	n/a	+/−	n/a	n/a	(−)	n/a
73.	Foal_25	Mare	86 d	n/a	n/a	n/a	n/a	n/a	n/a	(−)	n/a	(−)	n/a	n/a
74.	Stallion_14	Stallion	105 d	n/a	n/a	n/a	n/a	n/a	n/a	(−)	n/a	n/a	n/a	n/a
75.	Stallion_15	Stallion	19 y 5 m	n/a	n/a	n/a	n/a	n/a	n/a	(−)	n/a	n/a	n/a	(−)
76.	Mare_34	Mare	4 y 3 m	n/a	n/a	n/a	n/a	n/a	n/a	(−)	n/a	n/a	(−)	n/a

Interpretation of CT values: CT < 20 = ++++; CT 20.1–25 = +++; CT 25.1–30 = ++; CT 30.1–36 = +; CT 36.1–38.9 = +/−; CT > 39 = Negative (−). ^a^ Nasal swab samples from which EHV-4 virus have been isolated; n/a Not tested.

**Table 2:** Summary of VNT and glycoprotein G (gG) peptide ELISA results in paired serum samples. VNT—virus neutralization test; ELISA—enzyme linked immunosorbent assay.

Animals	Number of Samples	VNT				ELISA			
		2nd Week		9th Week		2nd Week		9th Week	
		Positive	Negative	Positive	Negative	Positive	Negative	Positive	Negative
**Foals**	24	0	24	3	21	24	0	24	0
**Mares**	24	24	0	24	0	24	0	24	0
**Total**	48	24	24	27	21	48	0	48	0

## Supplementary Materials

The following are available online at https://www.mdpi.com/article/10.3390/pathogens10070810/s1, Figure S1: Phylogenetic tree constructed by the maximum-likelihood method using full ORF30 gene (A), partial glycoprotein B [gB] gene (B) and gG gene (C) sequence of equine herpesvirus type 4 (EHV-4).
