# Supplementary material for: Equine Herpesvirus Type 4 (EHV-4) Outbreak in Germany: Virological, Serological, and Molecular Investigations
Source: Pathogens. 2021 Jun 25;10(7):810. doi: 10.3390/pathogens10070810 (PMC8308676; doi:10.3390/pathogens10070810)
Supplement: Supplementary file 1 [file pathogens-10-00810-s001.zip › pathogens-1232744-supplementary.pdf]

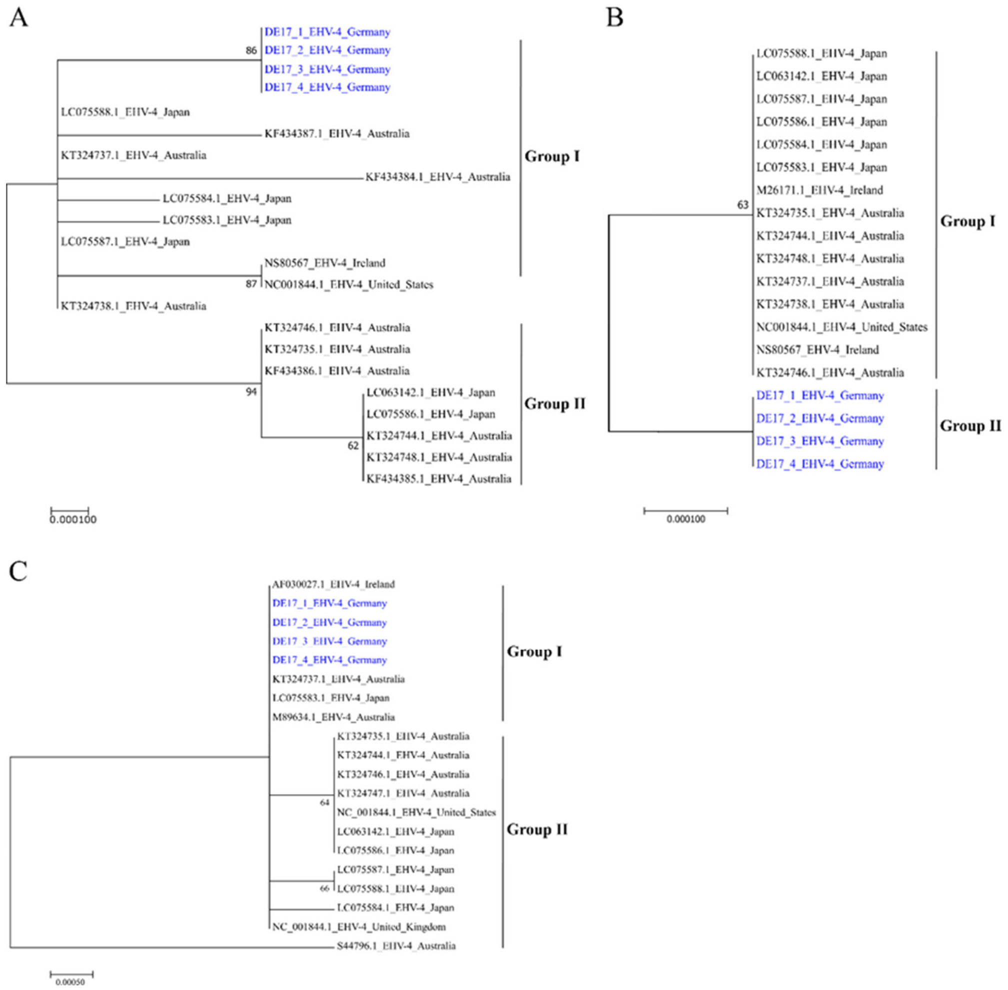


**Supplementary Figure S1.** Phylogenetic tree constructed by the maximum-likelihood method using full ORF30 gene (A), partial glycoprotein B [gB] gene (B) and gG gene (C) sequence of equine herpesvirus type 4 (EHV-4). GenBank accession numbers are indicated with country of origin. EHV-4 sequences from current study were indicated in blue.
